# Sustainable Fragment Peptide Synthesis (SFPS): Leveraging Oxyma as a Dual Resin Cleavage–Peptide Coupling Agent

**DOI:** 10.1021/acsomega.6c02679

**Published:** 2026-06-10

**Authors:** Jan Pawlas

**Affiliations:** PolyPeptide, Limhamnsvägen 108, PO BOX 30089, 20061 Limhamn, Sweden

## Abstract

Coupling of fully protected peptide fragments (FPPFs) released off acid labile polymer supports such as 2-chlorotrityl chloride (CTC) resin is a powerful peptide synthesis approach used from R&D to manufacturing type 2 diabetes/obesity drugs on a ton scale. Nevertheless, cleavage of acid labile FPPF resins typically entails PFAS-classified TFA as a reagent and suspected carcinogen DCM as a solvent while necessitating laborious work-ups and isolations. Here, in an approach we term sustainable fragment peptide synthesis (SFPS), a cleavage of FPPFs off 2-chlorotrityl (2-CT) resin was carried out using coupling additive ethyl cyanohydroxyiminoacetate (Oxyma) in EtOAc. The cleaved FPPF was not isolated but rather coupled with another peptide resin using a carbodiimide as a coupler and Oxyma from the cleavage as an additive. Showcasing the power of SFPS, a two Cys­(Trt) containing FPPF resin formed by a process leveraging Oxyma as a dual cleavage–coupling agent was cyclized to a disulfide containing FPPF resin which upon an Oxyma-induced cleavage afforded a disulfide containing FPPF, opening the door to the world of modular SFPS as a means of accessing a variety of complex peptide architectures.

## Graphical abstract

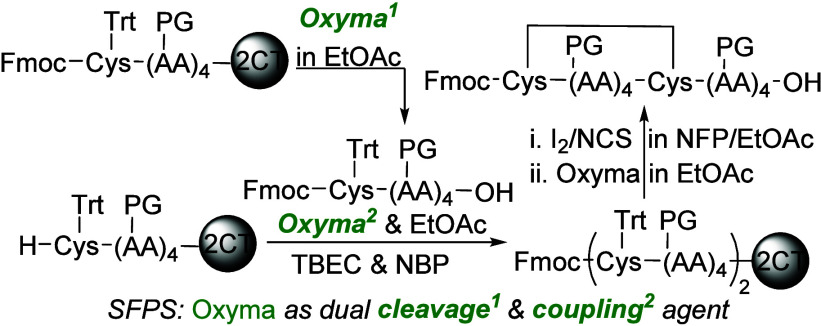

Commencing with Curtius dipeptide synthesis^1^ to inventing carbodiimides,^2^ SPPS,^3^ and Fmoc^4^ as the most consequential peptide couplers, the synthesis platform and protecting group (PG), respectively, the art of peptide synthesis encompasses a wide range of methods^5,6^ capable of accessing essentially any peptide irrespective of scale and complexity.^7^ Nevertheless, despite the prowess of contemporary peptide chemistry, a recent trend has emerged requiring efficiency and scalability to go hand in hand with the 12 principles of Green Chemistry put forth by Anastas.^8,9^ In fact, the need to reinvent peptide synthesis as a sustainable science and technology has been exacerbated by the significant increase in demand for large quantities of therapeutic peptides,^10^ in particular in the metabolic disease area,^11^ which, coupled with the reliance of current peptide synthesis methods on large quantities of hazardous materials,^12^ has led to concerns about producing active pharmaceutical ingredients (APIs) for peptide therapies of today and tomorrow in a sustainable and economically feasible manner.^13^ Indeed, responding to the aforementioned challenges facing the peptide manufacturing sector, ample approaches aimed at making peptides sustainably have been reported.^14^ In particular, with the widely used Fmoc/*t*-Bu SPPS^15^ hinging on large amounts of hazardous DMF as a solvent,^12,16^ various waste minimizing approaches^17^ together with less hazardous solvents,^18^ solvent mixtures,^19^ and even aqueous solutions^20^ for greener Fmoc/*t*-Bu SPPS^21^ have been put forth. Furthermore, an SPPS-linked concept gaining traction as a means of producing peptides sustainably is the coupling of fragments, with chemical^22^ and enzymatic^23^ ligations of unprotected fragments used in R&D extensively, with some recent advances indicating industrial viability.^24^ On the other hand, coupling of fully protected peptide fragments (FPPFs) has not only been utilized to synthesize peptides on a lab scale;^25^ from HIV fusion inhibitor enfuvirtide^26^ to GLP-1/GIP agonist tirzepatide,^27^ coupling FPPFs also has been championed as a sensible approach to produce therapeutic peptides in an industrial setting.^28^ Regarding FPPF synthesis, suitable solution phase methods have been reported,^29^ although releasing FPPFs from various polymer supports in general^30^ and using acid labile carriers such as 2-chlorotrityl chloride (CTC) resin^31^ in particular is the prevalent approach.

Nevertheless, the usefulness of FPPFs assembled on CTC resin is hampered by their reliance on PFAS substances,^32^ such as TFA,^25^ TFE,^31^ and HFIP^33^, as cleavage agents, as well as by hazardous DCM^25,31,33^ and DMF^34^ as solvents and by cumbersome workup procedures (Figure 1i.a).^35^ In this respect, it is worth noting that greener cleavages of FPPF 2-chlorotrityl (2-CT) resins have been reported, albeit their dependence on TFA remained unaddressed (Figure 1i.b).^19b,36^ In light of the lack of practical, sustainable methods for releasing FPPFs off 2-CT resins, we report that FPPF 2-CT resins can be cleaved in green solvents either by a Lewis acid (LA) such as FeCl_3_ or by a coupling additive such as Oxyma, the latter of which allows for using FPPFs directly in peptide couplings by adding a carbodiimide coupling agent, rendering Oxyma a dual resin cleavage–peptide coupling agent used within the concept of sustainable fragment peptide synthesis (SFPS) (Figure 1ii.c).

**Figure 1:**
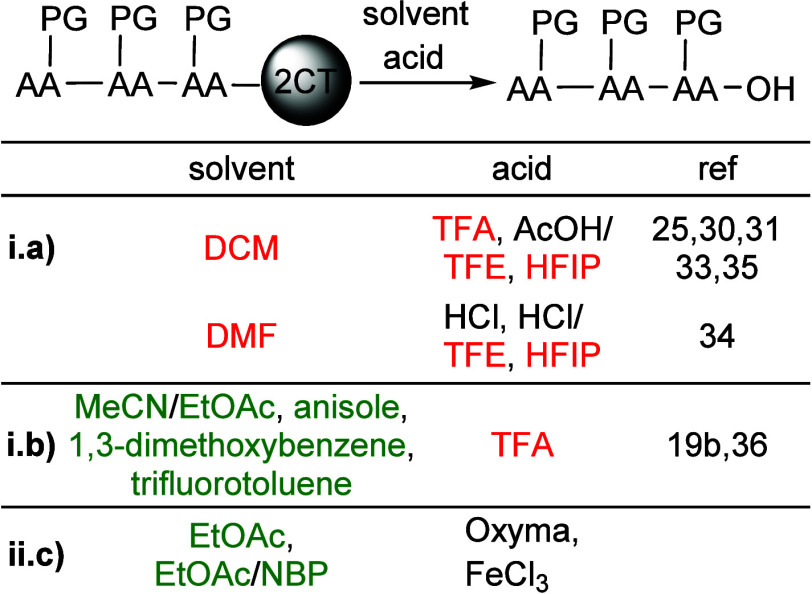
Acid-induced cleavages of FPPFs off 2-CT resins, (i) prior art: PFAS acids in DCM and DMF (a); TFA in green solvents (b); (ii) this work: Oxyma, FeCl_3_ in green solvents, SFPS (Scheme 2): Oxyma as a dual cleavage and coupling agent (c).

At the outset, we aimed to synthesize a suitable FPPF 2-CT resin as a substrate and to encompass the most common AA­(PG)­s used in Fmoc/*t*-Bu SPPS,^37^ pentapeptide resin comprising Ser­(*t-*Bu), Asp­(O*t*-Bu), Gln­(Trt), Lys­(Boc), and Cys­(Trt) functionalities was chosen. Aiming at carrying out sustainable peptide synthesis throughout the model resin was made in (*N*-butylpyrrolidone) NBP/EtOAc^19b,19^ by first coupling Fmoc-Ser­(*t*-Bu)-OH onto CTC resin^38^ followed by four AA coupling cycles using 4-methylpiperidine (4-MP)^39^ as the Fmoc removal base and 1-*t*-butyl-3-ethylcarbodiimide (TBEC)^40^/Oxyma^41^ as coupling agents, furnishing resin **2** in 62% yield (Scheme 1).

**Scheme 1:**
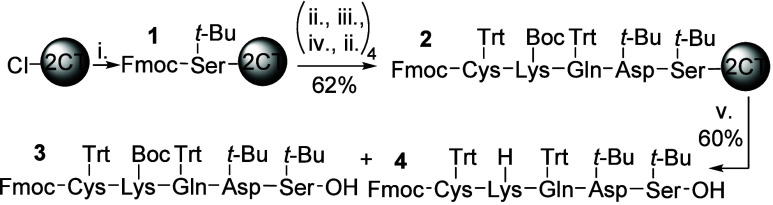
SPPS of Resin **2** in NBP/EtOAc (1:4) and Cleavage of **2** to Pentapeptide **3** by FeCl_3_ in EtOAc^a^

a Reagents and conditions: (i) 1 equiv Fmoc-Ser­(*t*-Bu)-OH/DIEA (1:2), 1 h, 30 °C; (ii) 10% 4-MP, 0.5 h, 30 °C; (iii) wash; (iv) 2 equiv AA/Oxyma/TBEC (1.0:1.0:1.3), 0.5 h, 30 °C; v. (a) 0.25% FeCl_3_/2.0%TIS in EtOAc, 1.0 h, rt; (b) heptane precipitation.

With **2** in hand, we turned to cleave FPPF **3** off the resin, first examining our recent protocol for cleaving pNZ-AA-2CT resins involving FeCl_3_ in EtOAc.^42^ Specifically, the cleavage of resin **2** using 0.25% FeCl_3_ in EtOAc with triisopropylsilane (TIS) as a scavenger afforded the target FPPF **3** in 60% yield (Scheme 1) although LC-MS of crude **3** also revealed the presence of 12% of des-Boc impurity **4** (Figure 2).

**Figure 2:**
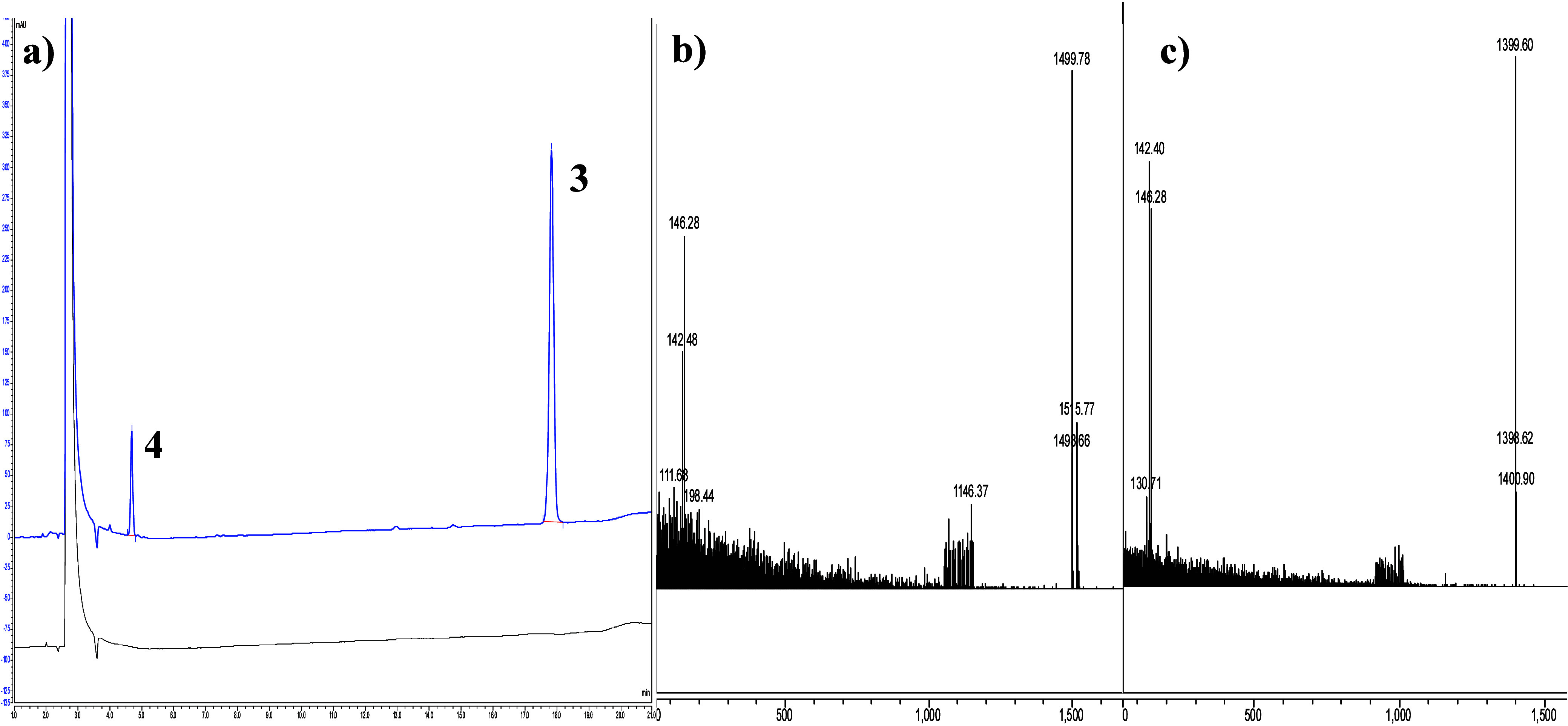
LC-MS of crude **3** from Scheme 1; (a) HPLC for crude from cleavage of resin **2** by FeCl_3_ in EtOAc; bottom, blank; top, product **3** containing 12% des-Boc byproduct **4**; (b) MS (ESI) *m*/*z* [M + H]^+^ for **3**, calcd 1498.70; found, 1498.66; (c) MS (ESI) *m*/*z* [M + H]^+^ for **4**, calcd 1398.65; found, 1398.62.

With the formation of des-Boc **4** in Scheme 1 during cleavage of resin **2** in agreement with FeCl_3_/DCM-induced Lys­(Boc) cleavages,^43^ we assessed further cleavage conditions aimed at suppressing **4** while attaining **3** in an adequate yield. As classical TFA in DCM afforded the highest amount of **3** after 3 h at rt (Table 1, entry 2) without any Lys­(Boc) cleavage (Figure S5), the entry 2 protocol was used as a benchmark for green cleavages of resin **2**. In this regard, we first attempted to suppress the loss of Boc encountered with FeCl_3_ in EtOAc by using more polar NBP/EtOAc employed in the SPPS of resin **2**. Gratifyingly, exposing **2** to FeCl_3_ in NBP/EtOAc over 16 h at 45 °C afforded **3** in an amount comparable to TFA/DCM (Table 1, entries 4, 7, and 10) without any concomitant Lys­(Boc) cleavage (Figure S5).

**Table 1:** Cleavage of Resin **2** to Pentapeptide **3** under Different Conditions^a^

entry	solvent	reagents	temperature	time (h)	**3** (%)^b^
1	DCM	1% TFA/2% TIS	rt	1.5	95
2	DCM	1% TFA/2% TIS	rt	3	100
3	DCM	1% TFA/2% TIS	rt	16	91
4	NBP/EtOAc 1:4	0.25% FeCl_3_/2% TIS	45 °C	1.5	37
5	NBP/EtOAc 1:4	0.2 M Oxyma/2% TIS	45 °C	1.5	2
6	NBP/EtOAc 1:4	0.25% FeCl_3_/0.2 M Oxyma/2% TIS	45 °C	1.5	43
7	NBP/EtOAc 1:4	0.25% FeCl_3_/2% TIS	45 °C	3	72
8	NBP/EtOAc 1:4	0.2 M Oxyma/2% TIS	45 °C	3	5
9	NBP/EtOAc 1:4	0.25% FeCl_3_/0.2 M Oxyma/2% TIS	45 °C	3	84
10	NBP/EtOAc 1:4	0.25% FeCl_3_/2% TIS	45 °C	16	>99
11	NBP/EtOAc 1:4	0.2 M Oxyma/2% TIS	45 °C	16	31
12	NBP/EtOAc 1:4	0.25% FeCl_3_/0.2 M Oxyma/2% TIS	45 °C	16	>99
13	NBP/EtOAc 1:4	0.2 M Oxyma/2% TIS	65 °C	1.5	44
14	NBP/EtOAc 1:4	0.2 M Oxyma/2% TIS	65 °C	16	>99
15	EtOAc	0.2 M Oxyma/2% TIS	45 °C	1.5	21
16	EtOAc	0.2 M Oxyma/2% TIS	45 °C	3	49
17	EtOAc	0.2 M Oxyma/2% TIS	45 °C	16	>99

a Carried out using 100 mg of resin in 1.0 mL of cleavage solution.

b Amount of **3** vs amount of **3** with 1% TFA in DCM after 3 h (entry 2).

Additionally, carrying out FeCl_3_-induced cleavage in the presence of Oxyma as a Bro̷nsted acid (BA) accelerated the rate of resin **3** cleavage (entries 6 and 9), indicating that BA–LA effects^44^ influencing the cleavage rate could have been at play. In fact, considering that Oxyma in DMF caused leaching of 2-CT resin bound peptides,^45^ we envisaged that the cleavage of FPPFs off 2-CT resin solely by coupling additives could be realized. Nevertheless, while the cleavage of **2** by Oxyma in NBP/EtOAc at 45 °C did lead to the formation of **3**, the rate was not sufficient to exploit this protocol preparatively (entries 5, 8, and 11). Yet, upon attempting to increase the rate of Oxyma-induced cleavage by using less polar EtOAc as solvent, **3** was afforded in an amount comparable to the TFA/DCM reference cleavage (entry 17). In fact, upon increasing temperature to 65 °C, adequate Oxyma-induced cleavage of resin **3** was attained in NBP/EtOAc as well (entries 13 and 14), and taken together, the cleavages of resin **2** in Table 1 outline various useful sustainable means for releasing FPPFs from 2-CT resin. With regard to the amounts of **3** cleaved off resin **2**, it is worth noting that for four of the cleavages in Table 1 (entries 10, 12, 14, and 17), a higher concentration of **3** in the final cleavage solution was obtained than for the entry 2 reference TFA/DCM cleavage. For these cleavages, the amount of **3** cleaved off the resin **2** was assigned as >99% vs the entry 2 reference cleavage. Nevertheless, while it is conceivable that in these instances a higher amount of **3** was in fact cleaved off resin **2** than in the reference cleavage, we have also noted that for these cleavages which were carried out at an elevated temperature for 16 h (typically overnight), a slightly decreased volume (∼10–15%) in the cleavage reactor (a fritted syringe sealed with a plastic piston) was observed, conceivably caused by slight EtOAc evaporation taking place over the course of the cleavage. Consequently, this decrease in the volume of the cleavage solution may have influenced the concentration of **3** in the final cleavage solution, and in future studies aimed at the advancement of sustainable peptide resin cleavages, experiments will be carried out to specifically evaluate all parameters impacting the amount of **3** in cleavage solutions for cleavages carried out for prolonged time at an elevated temperature. In addition, it is worth noting that in all acid-induced cleavages of resin **2** in Table 1 TIS (2%) was used as an additive. In this regard, while further studies will be required to determine the role TIS or any other scavengers play in sustainable cleavages of acid labile resins such as **2**, in keeping with our previous assessment of on-resin removal of trityl groups,^19b^ we propose that the primary role of silane scavenger in Table 1 cleavages is to ensure that the 2-CT resin cation formed during the cleavage is adequately quenched, preventing any deleterious side reactions involving the resin cation from taking place throughout the course of the cleavage of **2**. On the other hand, with regards to the role of Oxyma during the cleavages of peptides off 2-chlorotrityl resin discussed herein, in keeping with the findings of Albericio and co-workers who reported that coupling additives such as Oxyma and HOBt may cause leaching of peptides off 2-chlorotrityl resin upon prolonged standing in DMF,^45^ we propose that the weakly acidic Oxyma (p*K*_a_ ∼ 4.6 ^41^) behaves as any BA (e.g., the commonly used TFA or HFIP) in that it promotes acidolytic cleavage of the ester bond between the solid support and the peptide. Moreover, while evaluating different aspects of FPPF resin cleavages such as the choice of LA, coupling additive and solvent will be explored in future studies, to showcase the power of SFPS we used **3** cleaved from resin **2** by Oxyma in a coupling with a resin bound FPPF, utilizing Oxyma from the cleavage as a coupling agent.

Specifically, to **3** and Oxyma in EtOAc, obtained according to Table 1, entry 17 and removed from the spent 2-CT resin, NBP and TBEC were added to afford a ∼1:4 NBP/EtOAc solution of the Oxyma active ester of **3**. It is worth noting that while the optimization of the choice of coupling reagents and protocols for the direct couplings of FPPFs from sustainable cleavages of FPPF resins remains a subject of further studies, for the purpose of assessing the feasibility of coupling of FPPF **3** with resin **5,** we utilized a protocol comprising a ∼30 min preactivation of **3** in the presence of TBEC and Oxyma which worked well as a coupling protocol for coupling Fmoc-AA-OHs in NBP/EtOAc during a recent SPPS of an ε-Lys GLP-1 analog.^19l^ This ester was in turn reacted with FPPF resin **5**, affording 10-mer FPPF resin **6** (Scheme 2). 10-mer resin **6** thus obtained was then cleaved by Oxyma, followed by isolating FPPF **7** in 44% yield from resin **5** and 83% purity (Figure S7). In fact, as all of the Oxyma used in the coupling between resin **5** and FPPF **3** stemmed from the cleavage of resin **2** to FPPF **3**, the feasibility of the concept of SFPS involving the use of Oxyma as a dual cleavage–coupling agent was thereby demonstrated. It is worth noting that although in principle, coupling of FPPF **3** with resin **5** induced solely by TBEC is conceivable, in practice carbodiimide couplings necessitate ≥ 20 mol % Oxyma to reach adequate rates,^19g^ substantiating thereby the essential role Oxyma from the cleavage of resin **2** played as a reagent in coupling of **3** with **5**. With regard to the risk of unitended Oxyma-induced peptide cleavage during the coupling of **3** with **5,** it is worth noting that while the Oxyma-induced cleavage of FPPF **3** off 2-CT peptide resin **2** in Scheme 2 was carried out in neat EtOAc in which the cleavage rate was suitably fast to be exploited preparatively (Table 1, entries 15–17), before the coupling of **3** with **5** NBP was added to the coupling mixture together with TBEC which had an appreciable beneficial effect on the stability of the ester linkage between the peptide and the 2-CT polymer support. Specifically, as can be seen in Table 1, entry 5, the loss of peptide after 1.5 h at 45 °C in 0.2 M Oxyma in NBP/EtOAc (1:4) was only ∼2%, compared to ∼21% of **3** released off peptide resin **2** after 1.5 h at 45 °C in 0.2 M Oxyma in EtOAc (Table 1, entry 15). In this regard, as the Oxyma-mediated coupling between **3** and **5** in Scheme 2 was carried out in NBP/EtOAc (1:4) for 1.0 h at 30 °C, the loss of peptide can be anticipated to be much lower than the ∼2% observed after 1.5 h at 45 °C in 0.2 M Oxyma in NBP/EtOAc (1:4) (Table 1, entry 5). Moreover, the addition of TBEC to the coupling mixture containing Oxyma, **3** and **5** in NBP/EtOAc had conceivably a positive effect on the stability of the ester linkage between peptides and 2-CT resin as well, as while carrying peptide couplings on 2-CT peptide resins shall always be approached with caution,^46a^ using diisopropylcarbodiimide as the peptide coupler together with an acidic additive HOBt (p*K*_a_ ∼ 4.6 ^41^), Ieronymaki et al. reported that long peptides can be assembled on CTC resin under microwave (MW) irradiation aided SPPS at 70 °C in good yields.^46b^ Nevertheless, while taking our results on the stability of peptide resin **2** in Oxyma in NBP/EtOAc in Table 1, entry 5 together with the earlier work on MW SPPS of 2-CT peptide resins^46b^ indicates that the loss of peptide during the coupling of FPPF **3** with 2-CT peptide resin **5** in the presence of Oxyma in NBP/EtOAc in Scheme 2 was minimal; in our future studies aimed at further evaluation of various aspects of our SFPS methodology, stability of acid labile peptide resins under various coupling conditions will be carried out. Furthermore, as racemization in peptide couplings is always a concern,^47^ in our future studies epimerization in SFPS couplings will be thoroughly evaluated. Next, in light of the significance of disulfide containing peptide therapeutics^48^ and to further elevate SFPS by extending its reach to complex peptides, we converted the two Cys­(Trt) containing resin **6** to -SS- containing resin **8** utilizing our recent NCS/I_2_ based^20d^ green disulfide forming protocol.^49,50^ Cleavage of **8** with Oxyma then afforded −SS– containing FPPF **9** in 37% yield from resin **5** (Scheme 2) and 89% purity (Figures 3 and S10), opening for using SFPS in synthesis of a variety of cyclic peptide architectures. With regard to the isolation of the −SS– containing FPPF **9**, it is worth noting that while after the first heptane precipitation of **9** the crude peptide contained ∼49% of Oxyma after the second precipitation, the amount of Oxyma was suppressed to ∼1% (see Figure S9). As the focus of our study was cleaving acid labile peptide resins and then engaging the protected peptides thus obtained directly in peptide couplings using Oxyma as a reagent, we have not pursued further finetuning of the Oxyma-induced cleavage/precipitation as a part of the current work. Instead, as this matter warrants to be thoroughly investigated, all aspects pertaining to TFA-free peptide resin cleavages/protected peptide isolations will be tackled as future subjects of inquiry.

**Scheme 2:**
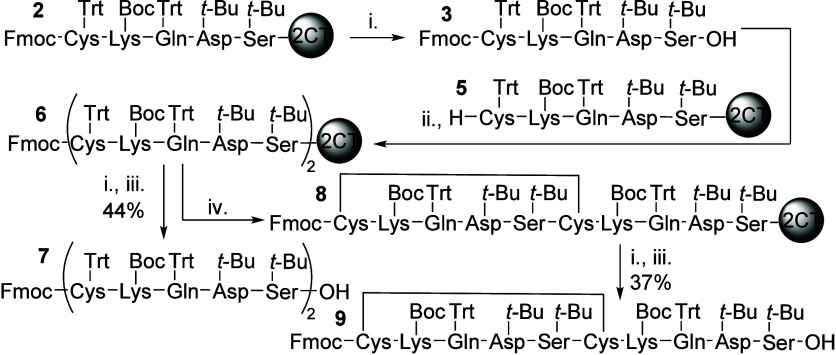
Oxyma as a Dual Cleavage and Coupling Agent in SFPS^a^

a Reagents and conditions: (i) 0.2 M Oxyma/2% TIS in EtOAc, 16 h, 45 °C; (ii) 2 equiv **3**/Oxyma in EtOAc from (i)/TBEC (1.0:4.0:5.2), NBP; 1.0 h, 30 °C; (iii) heptane precipitation; (iv) 25 mM I_2_/50 mM NCS in *N*-formylpyrrolidine (NFP)/EtOAc (1:1), 1.0 h, rt.

**Figure 3:**
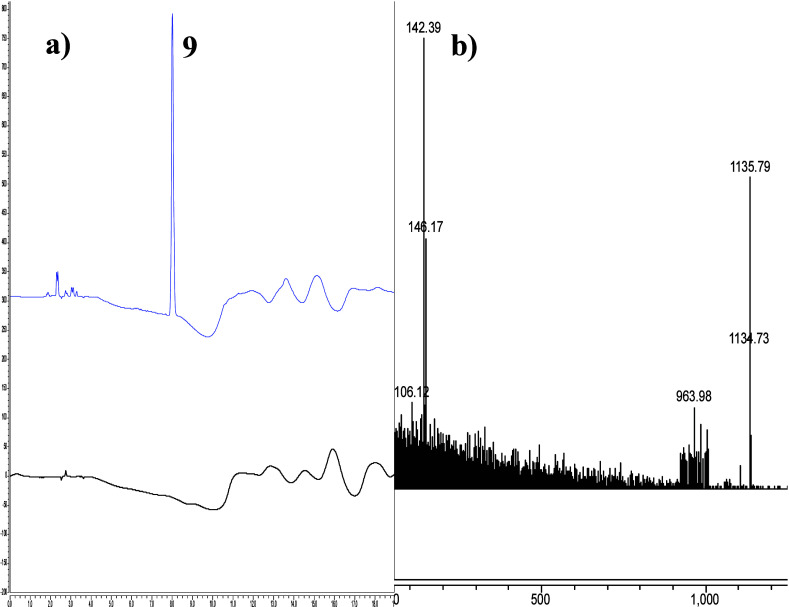
LC-MS of crude **9** from Scheme 2; (a) HPLC for crude **9**; bottom, blank; top, **9**; (b) MS (ESI) *m*/*z* [M + H]^+^ for **9**, calcd (*m* + *z*)/*z* (*z* = 2) 1135.55; found, 1135.79.

Finally, to extend SFPS beyond CTC resin-based FPPFs, we examined the cleavage of Fmoc-Leu-Sieber resin^30^ in which FeCl_3_ in EtOAc gave Fmoc-Leu-NH_2_ comparably to TFA in DCM (Figure S13),^51^ paving the way for using SFPS with a range of acid labile solid supports.^30,52^ In summary, we report the cleavage of an FPPF 2-CT resin containing AA­(PG)­s commonly used in Fmoc/*t*-Bu SPPS by coupling additive Oxyma in EtOAc. The FPPF thus obtained was not isolated but rather coupled with another FPPF 2-CT resin, utilizing a carbodiimide coupling agent along with Oxyma from the cleavage as an additive. The resulting bis Cys­(Trt) FPPF resin was cyclized, affording upon an Oxyma cleavage, an −SS– containing FPPF. We term the concept in which a coupling additive such as Oxyma serves in a dual resin cleavage–peptide coupling agent role *“*sustainable fragment peptide synthesis” (SFPS)*,*and owing to eliminating hazardous materials, circumventing tedious work-ups conventional fragment peptide syntheses rely on while granting facile access to complex peptide frameworks, we anticipate that the peptide fragment cleavage–coupling approach herein will alter the way in which peptides are synthesized from the lab to GMP manufacturing.

## Supplementary Material



## Data Availability

The data underlying this study are available in the article and its Supporting Information.

## Notes

Experimental and analytical data for SPPS, cleavage, and disulfide cyclization experiments (PDF)
