# Supplementary material for: Sustainable Fragment Peptide Synthesis (SFPS): Leveraging Oxyma as a Dual Resin Cleavage–Peptide Coupling Agent
Source: ACS Omega. 2026 Jun 10;11(24):35880–7. doi: 10.1021/acsomega.6c02679 (PMC13295053; doi:10.1021/acsomega.6c02679)
Supplement: Supplementary file 1 [file ao6c02679_si_001.pdf]

Electronic Supporting Information

Sustainable Fragment Peptide Synthesis (SFPS): Leveraging Oxyma as a Dual Resin Cleavage – Peptide Coupling Agent

Jan Pawlas[a,*]

[a]PolyPeptide, Limhamnsvägen 108, PO BOX 30089, 20061 Limhamn, Sweden

[*]Corresponding author. E-mail: jan.pawlas@polypeptide.com

Table of Contents

1. General information …………………………………………………………………… S2

2. SPPS of Fmoc-Cys(Trt)-Lys(Boc)-Gln(Trt)-Asp(O*t*-Bu)-Ser(*t*-Bu)-2CT resin………………S2

3. Cleavage of Fmoc-Cys(Trt)-Lys(Boc)-Gln(Trt)-Asp(O*t*-Bu)-Ser(*t*-Bu)-2CT resin using $FeCl_3$ in EtOAc……………………………………………………………………………………..S3

4. Assessment of cleavage of Fmoc-Cys(Trt)-Lys(Boc)-Gln(Trt)-Asp(O*t*-Bu)-Ser(*t*-Bu)-2CT resin under different conditions ……………………………………………………………..S6

5. Cleavage of Fmoc-Cys(Trt)-Lys(Boc)-Gln(Trt)-Asp(O*t*-Bu)-Ser(*t*-Bu)-2CT resin with Oxyma in EtOAc and coupling of the resulting Fmoc-Cys(Trt)-Lys(Boc)-Gln(Trt)-Asp(O*t*-Bu)-Ser(*t*-Bu)-OH and Oxyma with H-Cys(Trt)-Lys(Boc)-Gln(Trt)-Asp(O*t*-Bu)-Ser(*t*-Bu)-2CT using TBEC……………………………………………………………………………………..S8

6. Cleavage of Fmoc-Cys(Trt)-Lys(Boc)-Gln(Trt)-Asp(O*t*-Bu)-Ser(*t*-Bu)-Cys(Trt)-Lys(Boc)-Gln(Trt)-Asp(O*t*-Bu)-Ser(*t*-Bu)-2CT resin with Oxyma in EtOAc…………………………..….S8

7. On resin -SS- formation on Fmoc-Cys(Trt)-Lys(Boc)-Gln(Trt)-Asp(O*t*-Bu)-Ser(*t*-Bu)-Cys(Trt)-Lys(Boc)-Gln(Trt)-Asp(O*t*-Bu)-Ser(*t*-Bu)-2CT resin and cleavage of the resulting Fmoc-Cys(SS)-Lys(Boc)-Gln(Trt)-Asp(O*t*-Bu)-Ser(*t*-Bu)-Cys(SS)-Lys(Boc)-Gln(Trt)-Asp(O*t*-Bu)-Ser(*t*-Bu)-2CT resin with Oxyma in EtOAc ……………………………………………..…S11

8. Cleavage of Fmoc-Leu-Sieber resin with TFA in DCM and $FeCl_3$ in EtOAc ……………………………………………………………………………………………..S14

1. General information

All reagents, reactants, and solvents were from standard suppliers of raw materials for peptide synthesis and were used as such. All reactions which required heating were carried out by shaking in sealed fritted syringes (200 rpm) on an IKA® KS basic 130 apparatus or in an IKA KS 4000 ic control apparatus at the stated temperature for the stated time. Reactions which required shaking at rt were shaken at the stated 200 rpm using Heidolph Rotamax 120 shaker. LC-MS analyses were performed on a Thermoscientific MSQ Plus in a positive mode (ESI) coupled with Dionex UltiMate 3000. The specific LC conditions are described separately for each section of this ESI.

2. SPPS of Fmoc-Cys(Trt)-Lys(Boc)-Gln(Trt)-Asp(Ot-Bu)-Ser(t-Bu)-2CT resin

Experimental:

3.00 g of 1.0 – 1.6M CTC resin was weighed into a fritted syringe. Next, 1.15 g (3.0 mmol) Fmoc-Ser(*t*-Bu)-OH was added, followed by adding 15.0 mL NBP/EtOAc (1:4) and 1.05 mL (6.0 mmol) DIEA, sealing the syringe and shaking the resulting reaction mixture for 1 h at 30 °C. Next, the reaction mixture was quenched by adding 1.0 mL MeOH followed by shaking for 10 min at 30 °C and draining the resin. The resin was washed with 3 x 15 mL NBP/EtOAc (1:4) after which four AA coupling cycles were carried out as follows:

i) removal of the Fmoc group from the resin using 20.0 mL 10% 4-MP (v/v) in NBP/EtOAc (1:4) for 30 min at 30 °C.

ii) resin wash with 5 x 15 mL NBP/EtOAc (1:4)

iii) AA coupling; 6.0 mmol of AA and 0.85 g (6.0 mmol) Oxyma were dissolved in NBP/EtOAc (1:4) at 30 °C to which 1.06 mL (7.8 mmol) TBEC[1] was added and the resulting solution was preactivated by shaking at 30 °C for 30 min before adding the preactivated AA mixture to the Fmoc removed, washed resin. The coupling was then carried out by shaking at 30 °C for 30 min followed by draining the syringe. AAs used: 1st AA cycle, 2.47 g of Fmoc-Asp(O*t*-Bu)-OH; 2nd AA cycle, 3.66 g of Fmoc-Gln(Trt)-OH; 3rd AA cycle, 2.81 g of Fmoc-Lys(Boc)-OH; 4th AA cycle, 3.51 g of Fmoc-Cys(Trt)-OH.

iv) resin wash with 5 x 15 mL NBP/EtOAc (1:4)

Finally, the resin was washed with 3 x 15 mL *i*-PrOH and dried to constant weight in vacuo affording 7.75 g of Fmoc-Cys(Trt)-Lys(Boc)-Gln(Trt)-Asp(Ot-Bu)-Ser(t-Bu)-2CT resin. As the Fmoc content on the resin using a previously reported Fmoc quantification method[2] was determined to be 0.24M (Figure S1) the amount of the resin was 1.86 mmol, i.e. the yield of Fmoc-Cys(Trt)-Lys(Boc)-Gln(Trt)-Asp(Ot-Bu)-Ser(t-Bu)-2CT resin **2** was 62% (based on the amount of Fmoc-Ser(*t*-Bu)-OH used).

Analytical:

LC-MS analyses were performed on a Thermoscientific MSQ Plus in a positive mode (ESI) coupled with Dionex UltiMate 3000. HPLC conditions were as follows: SelectPeptide CSH C18, 130Å, 150 x 4.6mm, 2.5 μm column, TFA/H_2O (0.1:100, A), TFA/ MeCN (0.1:100, B) as buffers, 10% B to 90% B over 15 min gradient, flow rate = 0.6 mL min^{-1}, detection at l = 294 nm and column temperature 30 °C.

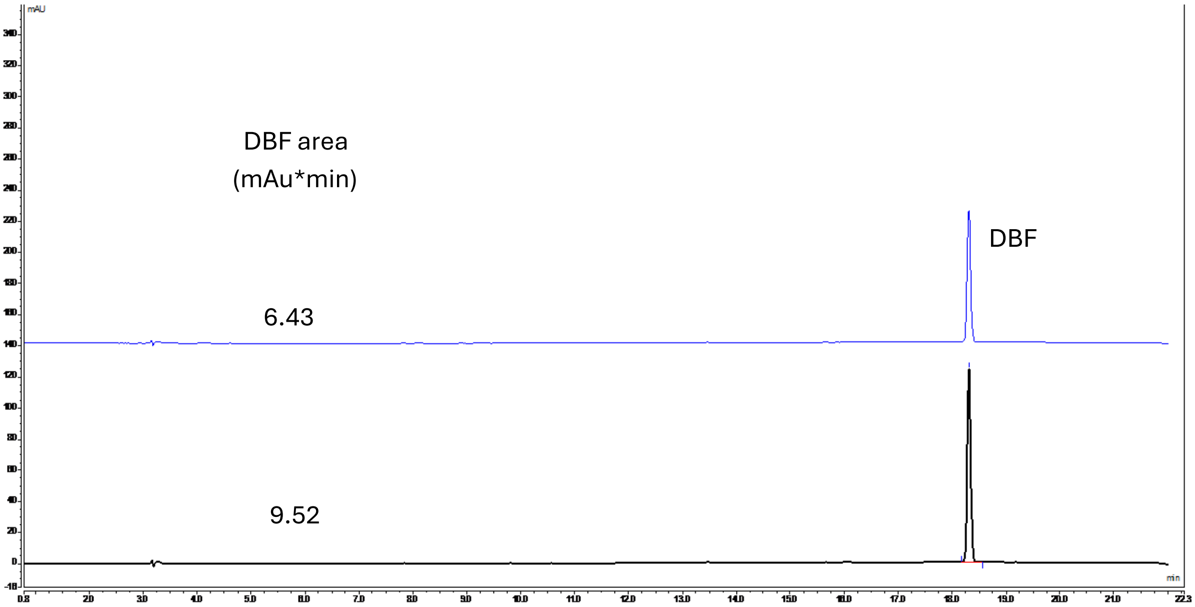

Figure S1. Overlay of LC-MS chromatograms for DBF peaks obtained by treatment of Fmoc-Cys(Trt)-Lys(Boc)-Gln(Trt)-Asp(Ot-Bu)-Ser(t-Bu)-2CT resin with 2% DBU in DMF; bottom, DBF peak for 0.35M Fmoc-Gly-MBH reference resin; top, DBF peak for Fmoc-Cys(Trt)-Lys(Boc)-Gln(Trt)-Asp(Ot-Bu)-Ser(t-Bu)-2CT resin **2**, determined to be 0.24M by comparing the area of DBF peak of the Fmoc-Cys(Trt)-Lys(Boc)-Gln(Trt)-Asp(Ot-Bu)-Ser(t-Bu)-2CT resin **2** with the DBF peak for 0.35M Fmoc-Gly-MBH reference resin.

3. Cleavage of Fmoc-Cys(Trt)-Lys(Boc)-Gln(Trt)-Asp(Ot-Bu)-Ser(t-Bu)-2CT resin using $FeCl_3$ in EtOAc

Experimental:

100 mg (0.024 mmol) Fmoc-Cys(Trt)-Lys(Boc)-Gln(Trt)-Asp(Ot-Bu)-Ser(t-Bu)-2CT resin **2** synthesized in section 2 of this ESI was weighed into a fritted syringe to which 2.5 mg $FeCl_3$ was added, followed by adding 977.5 μL EtOAc and 20 μL TIS. The syringe was sealed and

the resulting cleavage solution was shaken at rt for 1 h upon which the supernate was filtered off and the crude peptide was precipitated by addition of 30.0 mL heptane followed by centrifugation. The isolated crude peptide was again redissolved in 1.0 mL EtOAc and precipitated by addition of 30.0 mL heptane followed by centrifugation. The peptide product thus obtained was dried to constant weight in vacuo, affording 21.6 mg (60 %) of Fmoc-Cys(Trt)-Lys(Boc)-Gln(Trt)-Asp(Ot-Bu)-Ser(t-Bu)-OH **3** a sample of which was dissolved in DMF at 2 mg mL^{-1} and analyzed by LC-MS (Figures S2–4).

Analytical:

LC-MS analyses were performed on a Thermoscientific MSQ Plus in a positive mode (ESI) coupled with Dionex UltiMate 3000. HPLC conditions were as follows: SelectPeptide CSH C18, 130Å, 150 x 4.6mm, 2.5 μm column, TFA/H_2O (0.1:100, A), TFA/ MeCN (0.1:100, B) as buffers, 80% B to 90% B over 15 min gradient, flow rate = 0.6 mL min^{-1}, detection at I = 220 nm and column temperature 30 °C.

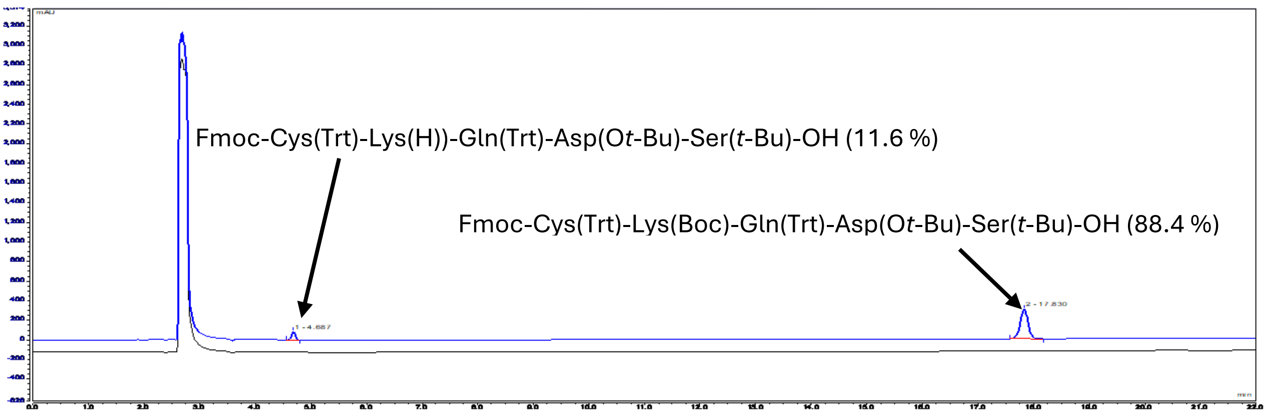

Figure S2. LC-MS chromatogram for Fmoc-Cys(Trt)-Lys(Boc)-Gln(Trt)-Asp(O*t*-Bu)-Ser(*t*-Bu)-OH **3** obtained by cleavage of Fmoc-Cys(Trt)-Lys(Boc)-Gln(Trt)-Asp(OtBu)-Ser(tBu)-2CT resin **2** by $FeCl_3$ in EtOAc; bottom, blank (DMF); top, crude Fmoc-Cys(Trt)-Lys(Boc)-Gln(Trt)-Asp(O*t*-Bu)-Ser(*t*-Bu)-OH **3**.

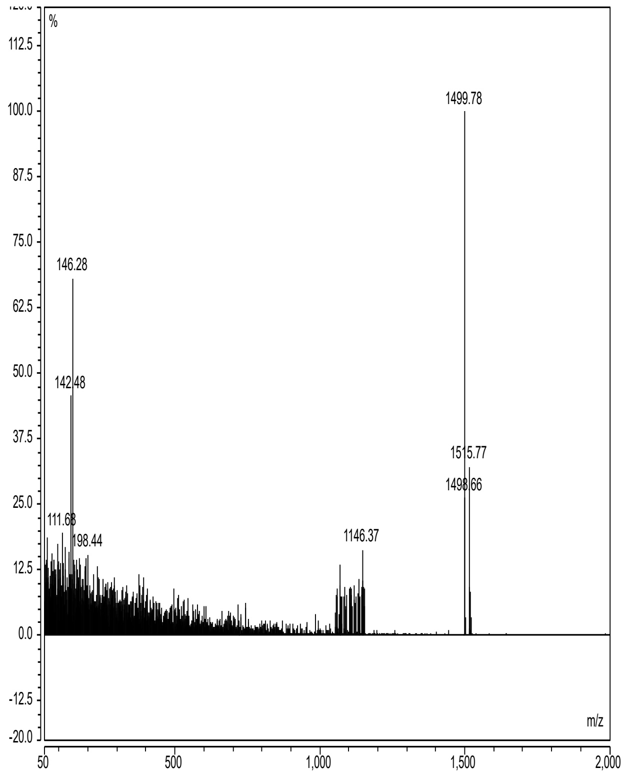

Figure S3. MS (ESI) m/z $[M+H]^+$ for Fmoc-Cys(Trt)-Lys(Boc)-Gln(Trt)-Asp(O*t*-Bu)-Ser(*t*-Bu)-OH **3** obtained by $FeCl_3$ in EtOAc cleavage of Fmoc-Cys(Trt)-Lys(Boc)-Gln(Trt)-Asp(O*t*-Bu)-Ser(*t*-Bu)-2CT resin **2**, calcd 1498.70; found, 1498.66.

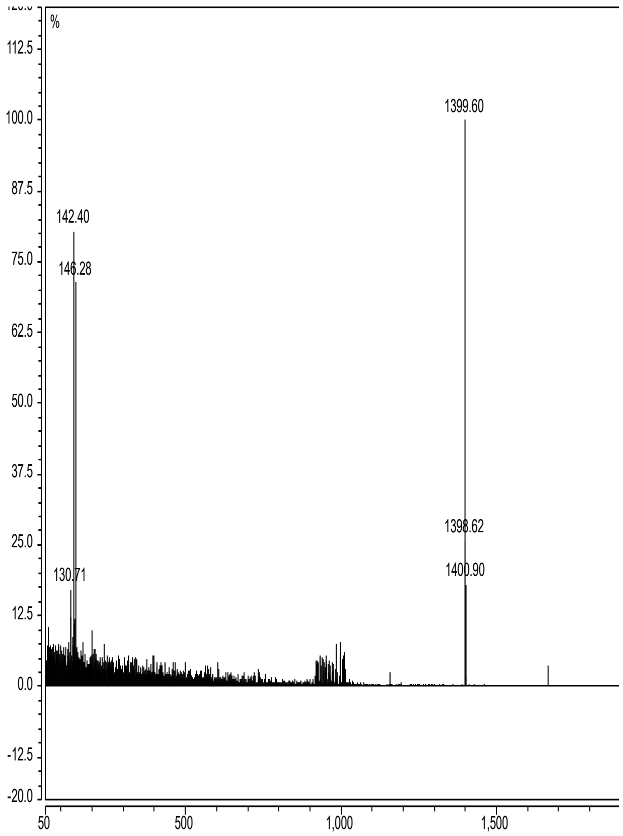

Figure S4. MS (ESI) m/z $[M+H]^+$ for Fmoc-Cys(Trt)-Lys(H)-Gln(Trt)-Asp(O*t*-Bu)-Ser(*t*-Bu)-OH byproduct **4** obtained by $FeCl_3$ in EtOAc cleavage of Fmoc-Cys(Trt)-Lys(Boc)-Gln(Trt)-Asp(O*t*-Bu)-Ser(*t*-Bu)-2CT resin **2**, calcd 1398.65; found, 1398.62.

4. Assessment of cleavage of Fmoc-Cys(Trt)-Lys(Boc)-Gln(Trt)-Asp(O*t*-Bu)-Ser(*t*-Bu)-2CT resin under different conditions

Experimental:

For all Fmoc-Cys(Trt)-Lys(Boc)-Gln(Trt)-Asp(O*t*-Bu)-Ser(*t*-Bu)-2CT resin **2** cleavage runs in Table 1, 100 mg (0.024 mmol) of Fmoc-Cys(Trt)-Lys(Boc)-Gln(Trt)-Asp(O*t*-Bu)-Ser(*t*-Bu)-2CT resin **2** synthesized in section 2 of this ESI was weighed into a fritted syringe. Next, starting materials and solvents were added to the syringes as described in entries of Table 1, followed by sealing the syringes and shaking them for times stated in entries of Table 1 at temperatures stated in entries of Table 1 after which a 50.0 µL aliquot of each cleavage mixture was added to 1.0 mL DMF. The resulting solutions of Fmoc-Cys(Trt)-Lys(Boc)-Gln(Trt)-Asp(O*t*-Bu)-Ser(*t*-Bu)-OH **3** were analyzed by LC-MS (Figure S5), determining the amounts of Fmoc-Cys(Trt)-Lys(Boc)-Gln(Trt)-Asp(O*t*-Bu)-Ser(*t*-Bu)-OH released off the resin by measuring the areas of Fmoc-Cys(Trt)-Lys(Boc)-Gln(Trt)-Asp(O*t*-Bu)-Ser(*t*-Bu)-OH for each run vs the amount of

Fmoc-Cys(Trt)-Lys(Boc)-Gln(Trt)-Asp(O*t*-Bu)-Ser(*t*-Bu)-OH released off the resin using 1% TFA/2% TIS in DCM after 3 h (Table 1, entry 2) .

Analytical:

LC-MS analyses were performed on a Thermoscientific MSQ Plus in a positive mode (ESI) coupled with Dionex UltiMate 3000. HPLC conditions were as follows: SelectPeptide CSH C18, 130Å, 150 x 4.6mm, 2.5 μm column, TFA/H_2O (0.1:100, A), TFA/ MeCN (0.1:100, B) as buffers, 80% B to 90% B over 15 min gradient, flow rate = 0.6 mL min^{-1}, detection at l = 220 nm and column temperature 30 °C.

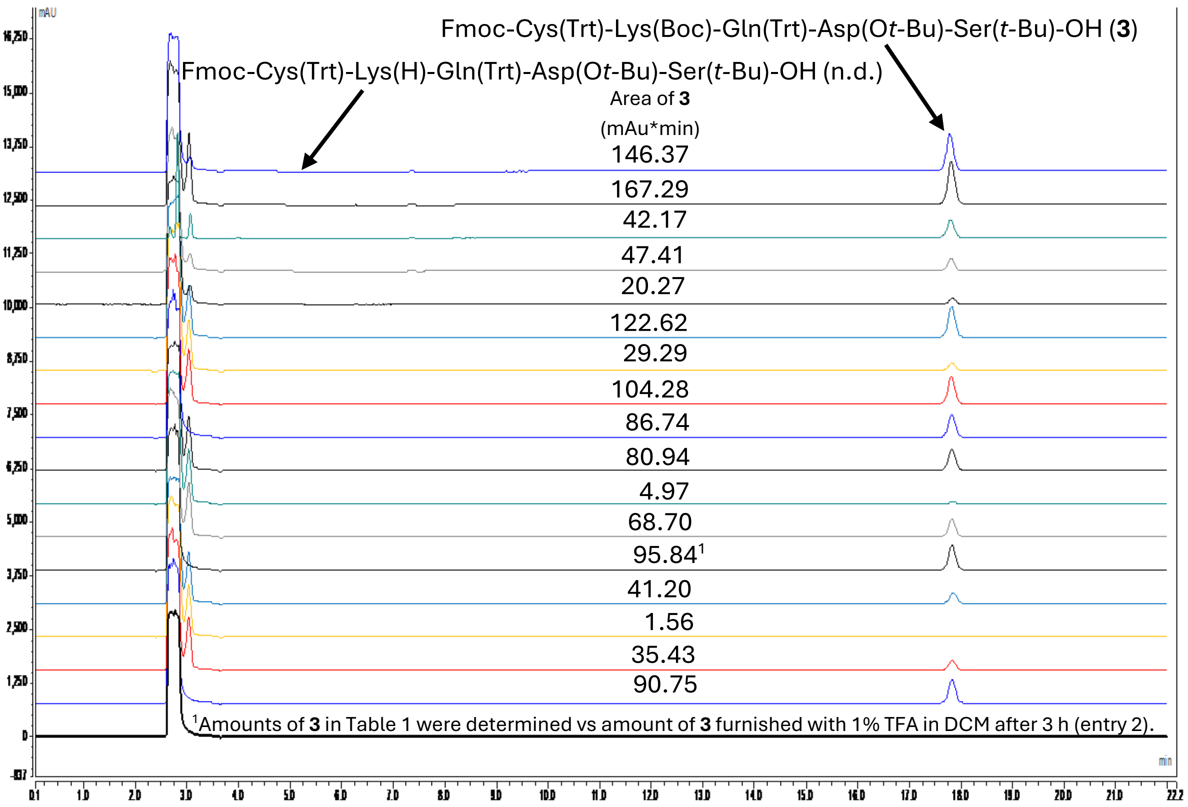

Figure S5. Overlay of LC-MS chromatograms for Fmoc-Cys(Trt)-Lys(Boc)-Gln(Trt)-Asp(O*t*-Bu)-Ser(*t*-Bu)-OH **3** obtained by cleavage of Fmoc-Cys(Trt)-Lys(Boc)-Gln(Trt)-Asp(OtBu)-Ser(tBu)-2CT resin **2** under different conditions; from bottom to top, i. blank (DMF); ii. crude **3** for Table 1, entry 1; iii. crude **3** for Table 1, entry 4; iv. crude **3** for Table 1, entry 5; v. crude **3** for Table 1, entry 6; vi. crude **3** for Table 1, entry 2; vii. crude **3** for Table 1, entry 7; viii. crude **3** for Table 1, entry 8; ix. crude **3** for Table 1, entry 9; x. crude **3** for Table 1, entry 3; xi. crude **3** for Table 1, entry 10; xii. crude **3** for Table 1, entry 11; xiii. crude **3** for Table 1, entry 12; xiv. crude **3** for Table 1, entry 15; xv. crude **3** for Table 1, entry 16; xvi. crude **3** for Table 1, entry 13; xvii. crude **3** for Table 1, entry 14; xviii. crude **3** for Table 1, entry 17.

5. Cleavage of Fmoc-Cys(Trt)-Lys(Boc)-Gln(Trt)-Asp(O*t*-Bu)-Ser(*t*-Bu)-2CT resin with Oxyma in EtOAc and coupling of the resulting Fmoc-Cys(Trt)-Lys(Boc)-Gln(Trt)-Asp(O*t*-Bu)-Ser(*t*-Bu)-OH and Oxyma with H-Cys(Trt)-Lys(Boc)-Gln(Trt)-Asp(O*t*-Bu)-Ser(*t*-Bu)-2CT using TBEC

Experimental:

1. Synthesis of H-Cys(Trt)-Lys(Boc)-Gln(Trt)-Asp(O*t*-Bu)-Ser(*t*-Bu)-2CT resin **5**

100 mg (0.024 mmol) of Fmoc-Cys(Trt)-Lys(Boc)-Gln(Trt)-Asp(O*t*-Bu)-Ser(*t*-Bu)-2CT resin **2** synthesized in section 2 of this ESI was weighed into a fritted syringe. Next, the Fmoc group was removed from the resin using 1.0 mL 10% 4-MP (v/v) in NBP/EtOAc (1:4) for 30 min at 30 °C followed by washing the resin with 5 x 2 mL NBP/EtOAc (1:4), 5 x 2 mL *i*-PrOH and drying the resin thus obtained to constant weight in vacuo.

2. Cleavage of Fmoc-Cys(Trt)-Lys(Boc)-Gln(Trt)-Asp(O*t*-Bu)-Ser(*t*-Bu)-2CT resin **2** with Oxyma in EtOAc

200 mg (0.048 mmol) Fmoc-Cys(Trt)-Lys(Boc)-Gln(Trt)-Asp(O*t*-Bu)-Ser(*t*-Bu)-2CT resin **2** synthesized in section 2 of this ESI was weighed into a fritted syringe. Next, according to the protocol in Table 1, entry 17, the resin was treated with 1.0 mL 0.2M Oxyma/2% TIS in EtOAc, the syringe was sealed and the resulting cleavage mixture was shaken at 45 °C for 16 h after which the supernate containing Fmoc-Cys(Trt)-Lys(Boc)-Gln(Trt)-Asp(O*t*-Bu)-Ser(*t*-Bu)-OH **3** and Oxyma in EtOAc was filtered off and its volume was adjusted to 1.0 mL by EtOAc.

3. Coupling of Fmoc-Cys(Trt)-Lys(Boc)-Gln(Trt)-Asp(O*t*-Bu)-Ser(*t*-Bu)-OH **3** with H-Cys(Trt)-Lys(Boc)-Gln(Trt)-Asp(O*t*-Bu)-Ser(*t*-Bu)-2CT resin **5**

To Fmoc-Cys(Trt)-Lys(Boc)-Gln(Trt)-Asp(O*t*-Bu)-Ser(*t*-Bu)-OH **3** and Oxyma in EtOAc synthesized in section 5.2 of this ESI was added 0.25 mL NBP and 34.0 µL (0.26 mmol) TBEC and the resulting mixture was activated at 30 °C for 0.5 h. The mixture thus obtained was then added to EtOAc swollen and drained H-Cys(Trt)-Lys(Boc)-Gln(Trt)-Asp(O*t*-Bu)-Ser(*t*-Bu)-2CT resin **5** synthesized in section 5.1 of this ESI. The resulting coupling mixture was shaken at 30 °C for 1.0 h after which the resulting resin **6** was drained and washed with 5 x 2 mL NBP/EtOAc (1:4) and 5 x 2 mL *i*-PrOH and dried to constant weight in vacuo.

6. Cleavage of Fmoc-Cys(Trt)-Lys(Boc)-Gln(Trt)-Asp(O*t*-Bu)-Ser(*t*-Bu)-Cys(Trt)-Lys(Boc)-Gln(Trt)-Asp(O*t*-Bu)-Ser(*t*-Bu)-2CT resin with Oxyma in EtOAc

Experimental:

In a fritted syringe, Fmoc-Cys(Trt)-Lys(Boc)-Gln(Trt)-Asp(O*t*-Bu)-Ser(*t*-Bu)-Cys(Trt)-Lys(Boc)-Gln(Trt)-Asp(O*t*-Bu)-Ser(*t*-Bu)-2CT resin **6** synthesized in section 5 of this ESI was exposed to 1.0 mL 0.2M Oxyma/2% TIS in EtOAc, at 45 °C for 16 h with shaking after which the supernate containing Fmoc-Cys(Trt)-Lys(Boc)-Gln(Trt)-Asp(O*t*-Bu)-Ser(*t*-Bu)-Cys(Trt)-

Lys(Boc)-Gln(Trt)-Asp(O*t*-Bu)-Ser(*t*-Bu)-OH **7** and Oxyma in EtOAc was filtered off and the crude peptide was precipitated by addition of 30.0 mL heptane followed by centrifugation. The isolated crude peptide was again redissolved in 1.0 mL EtOAc and precipitated by addition of 30.0 mL heptane followed by centrifugation. The peptide product thus obtained was dried to constant weight in vacuo, affording 29.1 mg (44 %) of Fmoc-Cys(Trt)-Lys(Boc)-Gln(Trt)-Asp(O*t*-Bu)-Ser(*t*-Bu)-Cys(Trt)-Lys(Boc)-Gln(Trt)-Asp(O*t*-Bu)-Ser(*t*-Bu)-OH **7** a sample of which was dissolved in DMF at 2 mg mL^{-1} and analyzed by LC-MS (Figures S7–8).

Analytical:

LC-MS analyses were performed on a Thermoscientific MSQ Plus in a positive mode (ESI) coupled with Dionex UltiMate 3000. HPLC conditions were as follows: SelectPeptide CSH C18, 130Å, 150 x 4.6mm, 2.5 μm column, TFA/H_2O (0.1:100, A), TFA/ MeCN (0.1:100, B) as buffers, 90% B to 98% B over 5 min gradient and 98% B for 13 min, flow rate = 0.6 mL min^{-1}, detection at l = 220 nm and column temperature 30 °C.

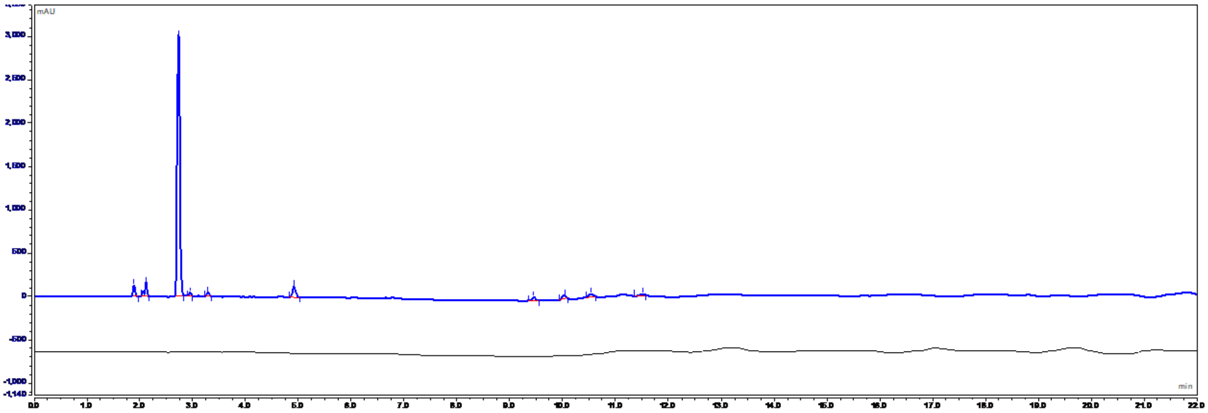

Figure S7. LC-MS chromatogram for Fmoc-Cys(Trt)-Lys(Boc)-Gln(Trt)-Asp(O*t*-Bu)-Ser(*t*-Bu)-Cys(Trt)-Lys(Boc)-Gln(Trt)-Asp(O*t*-Bu)-Ser(*t*-Bu)-OH **7** obtained by cleavage of Fmoc-Cys(Trt)-Lys(Boc)-Gln(Trt)-Asp(O*t*-Bu)-Ser(*t*-Bu)-Cys(Trt)-Lys(Boc)-Gln(Trt)-Asp(O*t*-Bu)-Ser(*t*-Bu)-2CT resin **6** with Oxyma in EtOAc; bottom, blank (MeCN); top, crude Fmoc-Cys(Trt)-Lys(Boc)-Gln(Trt)-Asp(O*t*-Bu)-Ser(*t*-Bu)-Cys(Trt)-Lys(Boc)-Gln(Trt)-Asp(O*t*-Bu)-Ser(*t*-Bu)-OH **7**.

Table S1. Area% for integrated peaks for LC-MS chromatogram for Fmoc-Cys(Trt)-Lys(Boc)-Gln(Trt)-Asp(O*t*-Bu)-Ser(*t*-Bu)-Cys(Trt)-Lys(Boc)-Gln(Trt)-Asp(O*t*-Bu)-Ser(*t*-Bu)-OH **7** obtained by cleavage of Fmoc-Cys(Trt)-Lys(Boc)-Gln(Trt)-Asp(O*t*-Bu)-Ser(*t*-Bu)-Cys(Trt)-Lys(Boc)-Gln(Trt)-Asp(O*t*-Bu)-Ser(*t*-Bu)-2CT resin **6** with Oxyma in EtOAc.

Peak no.	Ret.time (min)	Rel. area (%)	Area (mAU*min)	Height (mAU)
1	1.890	2.33	5.0766	127.31
2	2.120	3.53	7.6676	156.14
**3**	**2.733**	**82.92**	**180.3012**	**2997.16**
4	2.953	0.78	1.6942	36.04
5	3.290	1.00	2.1652	44.65
6	4.920	4.21	9.1556	129.31
7	9.457	1.62	3.5306	47.64
8	10.030	1.40	3.0353	40.37
9	10.540	1.44	3.1405	31.58
10	11.517	0.77	1.6744	18.67
Sum		100.00	217.4410	3628.87

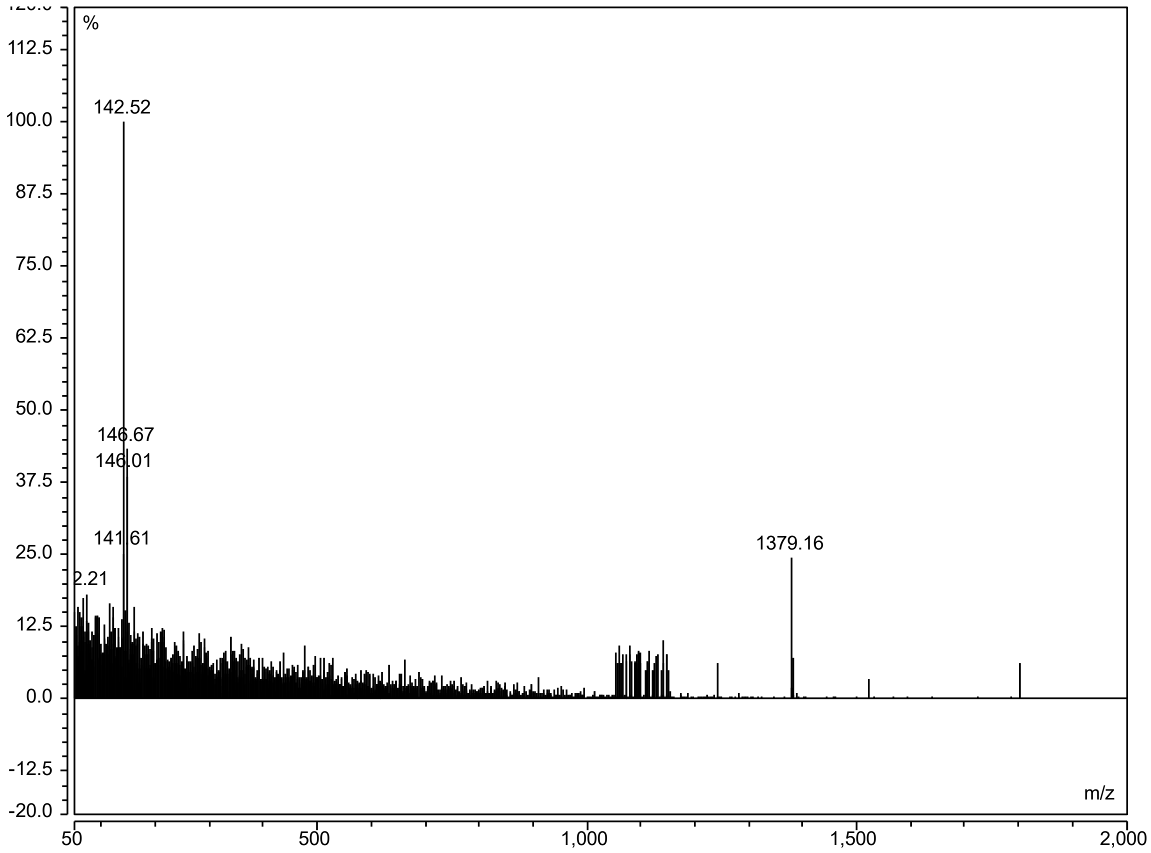

Figure S8. MS (ESI) m/z [M+H]+ for Fmoc-Cys(Trt)-Lys(Boc)-Gln(Trt)-Asp(O*t*-Bu)-Ser(*t*-Bu)-Cys(Trt)-Lys(Boc)-Gln(Trt)-Asp(O*t*-Bu)-Ser(*t*-Bu)-OH **7** cleaved from Fmoc-Cys(Trt)-Lys(Boc)-Gln(Trt)-Asp(O*t*-Bu)-Ser(t-Bu)-Cys(Trt)-Lys(Boc)-Gln(Trt)-Asp(O*t*-Bu)-Ser(*t*-Bu)-2CT resin **6** with Oxyma in EtOAc, calcd (m+z)/z (z=2) 1378.67; found, 1379.16.

7. On resin -SS- formation on Fmoc-Cys(Trt)-Lys(Boc)-Gln(Trt)-Asp(O*t*-Bu)-Ser(*t*-Bu)-Cys(Trt)-Lys(Boc)-Gln(Trt)-Asp(O*t*-Bu)-Ser(*t*-Bu)-2CT resin and cleavage of the resulting Fmoc-Cys(SS)-Lys(Boc)-Gln(Trt)-Asp(O*t*-Bu)-Ser(*t*-Bu)-Cys(SS)-Lys(Boc)-Gln(Trt)-Asp(O*t*-Bu)-Ser(*t*-Bu)-2CT resin with Oxyma in EtOAc

Experimental:

1) On resin -SS- formation on Fmoc-Cys(Trt)-Lys(Boc)-Gln(Trt)-Asp(O*t*-Bu)-Ser(*t*-Bu)-Cys(Trt)-Lys(Boc)-Gln(Trt)-Asp(O*t*-Bu)-Ser(*t*-Bu)-2CT resin **6**

In a fritted syringe, Fmoc-Cys(Trt)-Lys(Boc)-Gln(Trt)-Asp(O*t*-Bu)-Ser(*t*-Bu)-Cys(Trt)-Lys(Boc)-Gln(Trt)-Asp(O*t*-Bu)-Ser(*t*-Bu)-2CT resin **6** synthesized according to the protocol in section 5 of this ESI was exposed to a previously reported on-resin disulfide forming protocol[3] as follows: 25 mM I_2/50 mM NCS in NFP/EtOAc (1:1) was added to the resin, the syringe was sealed and the resulting disulfide forming reaction mixture was shaken at rt for 1 h, drained, washed with 5 x 2 mL NFP/EtOAc (1:1), 5 x 2 mL NBP/EtOAc (1:4), 5 x 2 mL 2-MeTHF a 5 x 2 mL *i*-PrOH and dried to constant weight in vacuo.

2) Cleavage of Fmoc-Cys(SS)-Lys(Boc)-Gln(Trt)-Asp(O*t*-Bu)-Ser(*t*-Bu)-Cys(SS)-Lys(Boc)-Gln(Trt)-Asp(O*t*-Bu)-Ser(*t*-Bu)-2CT resin **8** with Oxyma in EtOAc

In a fritted syringe, Fmoc-Cys(SS)-Lys(Boc)-Gln(Trt)-Asp(O*t*-Bu)-Ser(*t*-Bu)-Cys(SS)-Lys(Boc)-Gln(Trt)-Asp(O*t*-Bu)-Ser(*t*-Bu)-2CT resin **8** synthesized in section 7.1 of this ESI was exposed to 1.0 mL 0.2M Oxyma/2% TIS in EtOAc, at 45 °C for 16 h with shaking after which the supernate containing Fmoc-Cys(SS)-Lys(Boc)-Gln(Trt)-Asp(O*t*-Bu)-Ser(*t*-Bu)-Cys(SS)-Lys(Boc)-Gln(Trt)-Asp(O*t*-Bu)-Ser(*t*-Bu)-OH **9** and Oxyma in EtOAc was filtered off and the crude peptide was precipitated by addition of 30.0 mL heptane followed by centrifugation. The isolated crude peptide was again redissolved in 1.0 mL EtOAc and precipitated by addition of 30.0 mL heptane followed by centrifugation. The peptide product **9** thus obtained was dried to constant weight in vacuo, affording 20.1 mg (37 %) of Fmoc-Cys(SS)-Lys(Boc)-Gln(Trt)-Asp(O*t*-Bu)-Ser(*t*-Bu)-Cys(SS)-Lys(Boc)-Gln(Trt)-Asp(O*t*-Bu)-Ser(*t*-Bu)-OH **9** a sample of which was dissolved in DMF at 2 mg mL^{-1} and analyzed by LC-MS (Figures S10–S11).

Analytical:

LC-MS analyses were performed on a Thermoscientific MSQ Plus in a positive mode (ESI) coupled with Dionex UltiMate 3000. HPLC conditions were as follows: SelectPeptide CSH C18, 130Å, 150 x 4.6mm, 2.5 µm column, TFA/H_2O (0.1:100, A), TFA/ MeCN (0.1:100, B) as buffers, 90% B to 98% B over 5 min gradient and 98% B for 13 min, flow rate = 0.6 mL min^{-1}, detection at l = 220 nm and column temperature 30 °C.

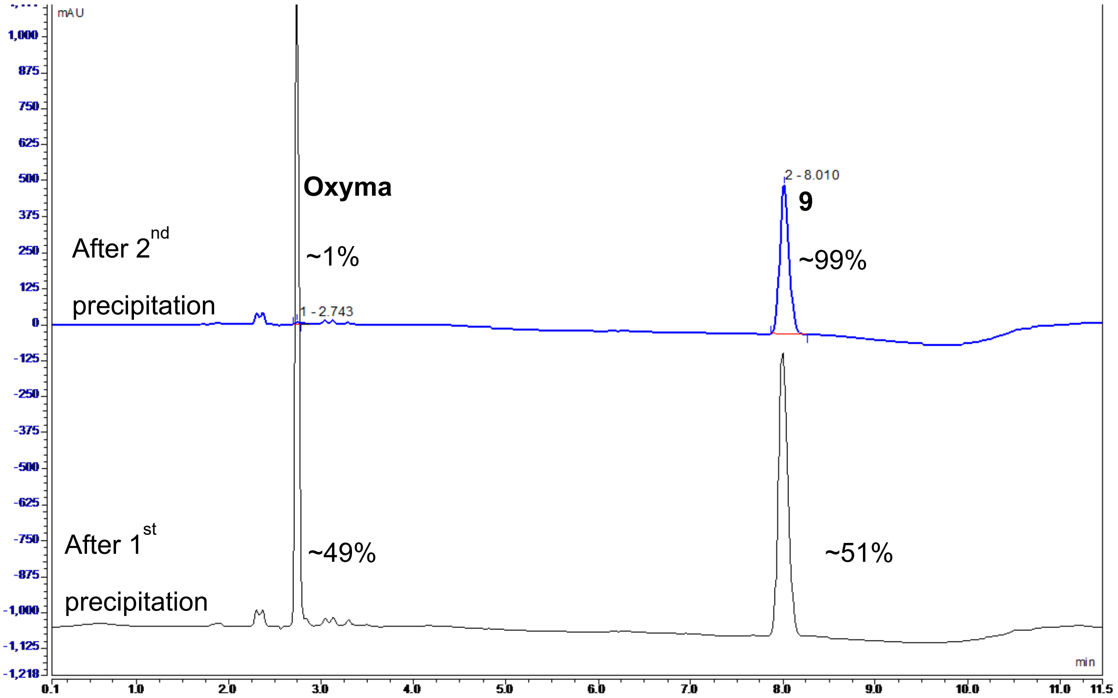

Figure S9. Overlay of LC-MS chromatograms for Fmoc-Cys(SS)-Lys(Boc)-Gln(Trt)-Asp(O*t*-Bu)-Ser(*t*-Bu)-Cys(SS)-Lys(Boc)-Gln(Trt)-Asp(O*t*-Bu)-Ser(*t*-Bu)-OH **9** obtained by cleavage

of Fmoc-Cys(SS)-Lys(Boc)-Gln(Trt)-Asp(O*t*-Bu)-Ser(*t*-Bu)-Cys(SS)-Lys(Boc)-Gln(Trt)-Asp(O*t*-Bu)-Ser(*t*-Bu)-2CT resin **8** with Oxyma in EtOAc; bottom, crude **9** after 1st heptane precipitation; top, crude **9** after 2nd heptane precipitation.

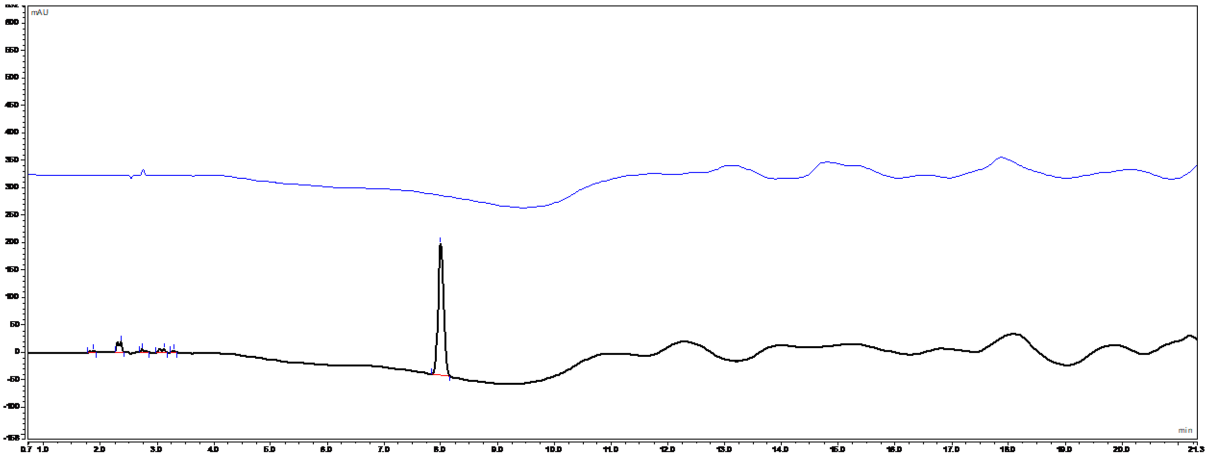

Figure S10. LC-MS chromatogram for Fmoc-Cys(SS)-Lys(Boc)-Gln(Trt)-Asp(O*t*-Bu)-Ser(*t*-Bu)-Cys(SS)-Lys(Boc)-Gln(Trt)-Asp(O*t*-Bu)-Ser(*t*-Bu)-OH **9** obtained by cleavage of Fmoc-Cys(SS)-Lys(Boc)-Gln(Trt)-Asp(O*t*-Bu)-Ser(*t*-Bu)-Cys(SS)-Lys(Boc)-Gln(Trt)-Asp(O*t*-Bu)-Ser(*t*-Bu)-2CT resin **8** with Oxyma in EtOAc; top, blank (MeCN); bottom, crude Fmoc-Cys(SS)-Lys(Boc)-Gln(Trt)-Asp(O*t*-Bu)-Ser(*t*-Bu)-Cys(SS)-Lys(Boc)-Gln(Trt)-Asp(O*t*-Bu)-Ser(*t*-Bu)-OH **9** after 2nd heptane precipitation.

Table S2. Area% for integrated peaks for LC-MS chromatogram for Fmoc-Cys(SS)-Lys(Boc)-Gln(Trt)-Asp(O*t*-Bu)-Ser(*t*-Bu)-Cys(SS)-Lys(Boc)-Gln(Trt)-Asp(O*t*-Bu)-Ser(*t*-Bu)-OH **9** obtained by cleavage of Fmoc-Cys(SS)-Lys(Boc)-Gln(Trt)-Asp(O*t*-Bu)-Ser(*t*-Bu)-Cys(SS)-Lys(Boc)-Gln(Trt)-Asp(O*t*-Bu)-Ser(*t*-Bu)-2CT resin **8** with Oxyma in EtOAc.

Peak no.	Ret.time (min)	Rel. area (%)	Area (mAU*min)	Height (mAU)
1	1.873	0.80	0.2395	3.28
2	2.363	5.69	1.7060	18.68
3	2.740	1.20	0.3601	4.82
4	3.120	2.27	0.6797	6.43

5	3.287	0.62	0.1856	3.76
**6**	**7.993**	**89.43**	**26.8331**	**239.44**
Sum		100.00	30.0041	276.40

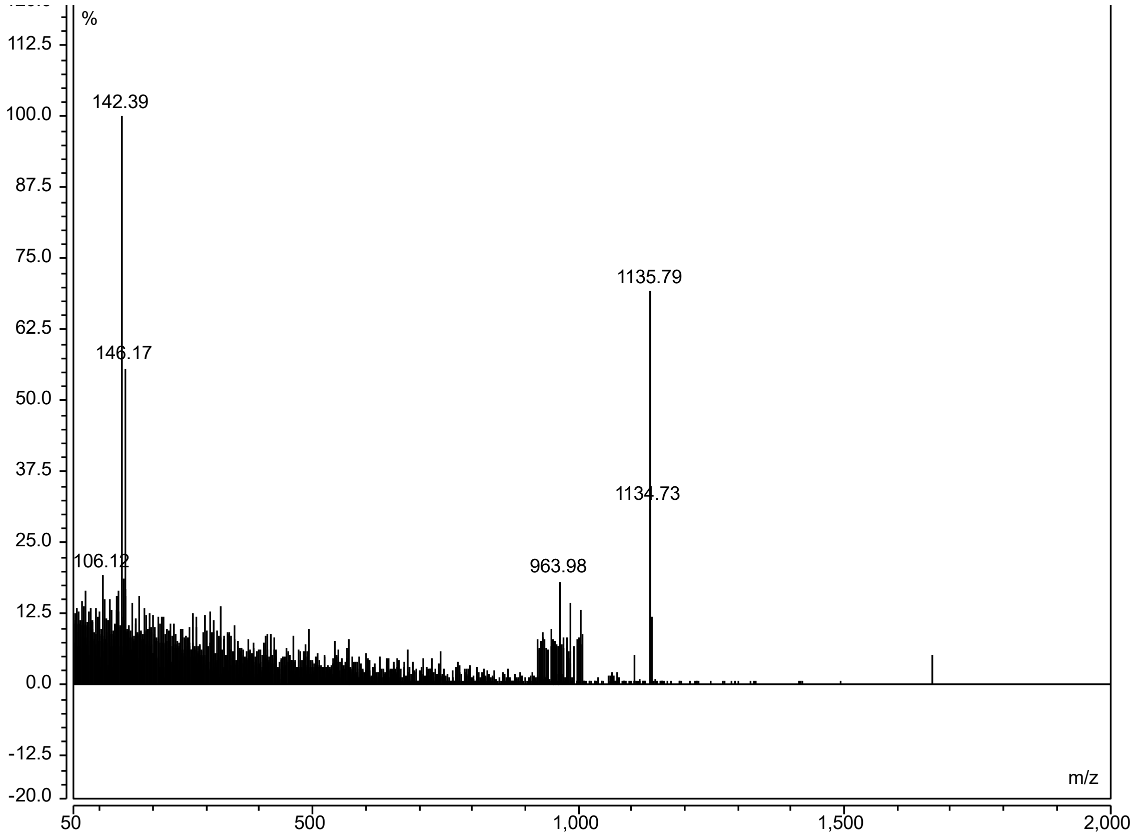

Figure S11. MS (ESI) m/z [M+H]+ for Fmoc-Cys(SS)-Lys(Boc)-Gln(Trt)-Asp(O*t*-Bu)-Ser(*t*-Bu)-Cys(SS)-Lys(Boc)-Gln(Trt)-Asp(O*t*-Bu)-Ser(*t*-Bu)-OH **9** cleaved from Fmoc-Cys(SS)-Lys(Boc)-Gln(Trt)-Asp(O*t*-Bu)-Ser(t-Bu)-Cys(SS)-Lys(Boc)-Gln(Trt)-Asp(O*t*-Bu)-Ser(*t*-Bu)-2CT resin **8** with Oxyma in EtOAc, calcd (m+z)/z (z=2) 1135.55; found, 1135.79.

8. Cleavage of Fmoc-Leu-Sieber resin with TFA in DCM and $FeCl_3$ in EtOAc

Experimental:

1) Synthesis of Fmoc-Leu-Sieber resin

1.0 g (0.70 mmol) of 0.7M Fmoc-Sieber resin was weighed into a fritted syringe after which the Fmoc group was removed from the resin using 20.0 mL 10% 4-MP (v/v) in NBP/EtOAc (1:4) for 30 min at 30 °C followed by a resin wash with 5 x 15 mL NBP/EtOAc (1:4). Next, 494.7 mg (1.4 mmol) of Fmoc-Leu-OH and 0.20 g (1.4 mmol) Oxyma were dissolved in

NBP/EtOAc (1:4) at 30 °C to which 0.23 mL (1.8 mmol) TBEC was added and the resulting solution was preactivated by shaking at 30 °C for 30 min before adding the preactivated AA mixture to the Fmoc removed, washed resin. The coupling was then carried out by shaking at 30 °C for 30 min followed by draining the syringe, washing the resin with 5 x 15 mL NBP/EtOAc and 3 x 15 mL *i*-PrOH and dried to constant weight in vacuo affording 1.11 g of Fmoc-Leu-Sieber resin. As the Fmoc content on the resin using a previously reported Fmoc quantification method[2] was determined to be 0.47M (Figure S12) the amount of the resin was 0.52 mmol, i.e. the yield of Fmoc-Leu-Sieber resin was 75% (based on the amount of Fmoc-Sieber resin used).

2) Cleavage of Fmoc-Leu-Sieber resin with TFA in DCM and $FeCl_3$ in EtOAc

Into two fritted syringes, 100.0 mg of Fmoc-Leu-Sieber resin synthesized in section 8.1 of this ESI was weighed in. Next, to the first syringe, 1.0 mL 2% TFA/TIS in DCM was added and to the second syringe 1.0 mL 0.25% $FeCl_3$/2% TIS in EtOAc were added. The syringes were sealed and shaken for 24 h at rt after which 50.0 µL aliquots of the reaction mixtures were diluted with 1.0 mL MeCN and analyzed by LC-MS vs 1.0 mg mL^{-1} Fmoc-Leu-OH as a reference standard (Figure S13). These LC-MS analyses revealed that

i) ~18.6 mg (>99% of theory) of Fmoc-Leu-NH_2 was cleaved off the Fmoc-Leu-Sieber resin using TFA in DCM as the cleavage agent

ii) ~14.3 mg (86% of theory) of Fmoc-Leu-NH_2 was cleaved off the Fmoc-Leu-Sieber resin using $FeCl_3$ in EtOAc as the cleavage agent

Analytical:

LC-MS analyses were performed on a Thermoscientific MSQ Plus in a positive mode (ESI) coupled with Dionex UltiMate 3000. HPLC conditions were as follows: SelectPeptide CSH C18, 130Å, 150 x 4.6mm, 2.5 µm column, TFA/H_2O (0.1:100, A), TFA/ MeCN (0.1:100, B) as buffers, 10% B to 90% B over 15 min gradient, flow rate = 0.6 mL min^{-1}, detection at l = 294 nm and column temperature 30 °C.

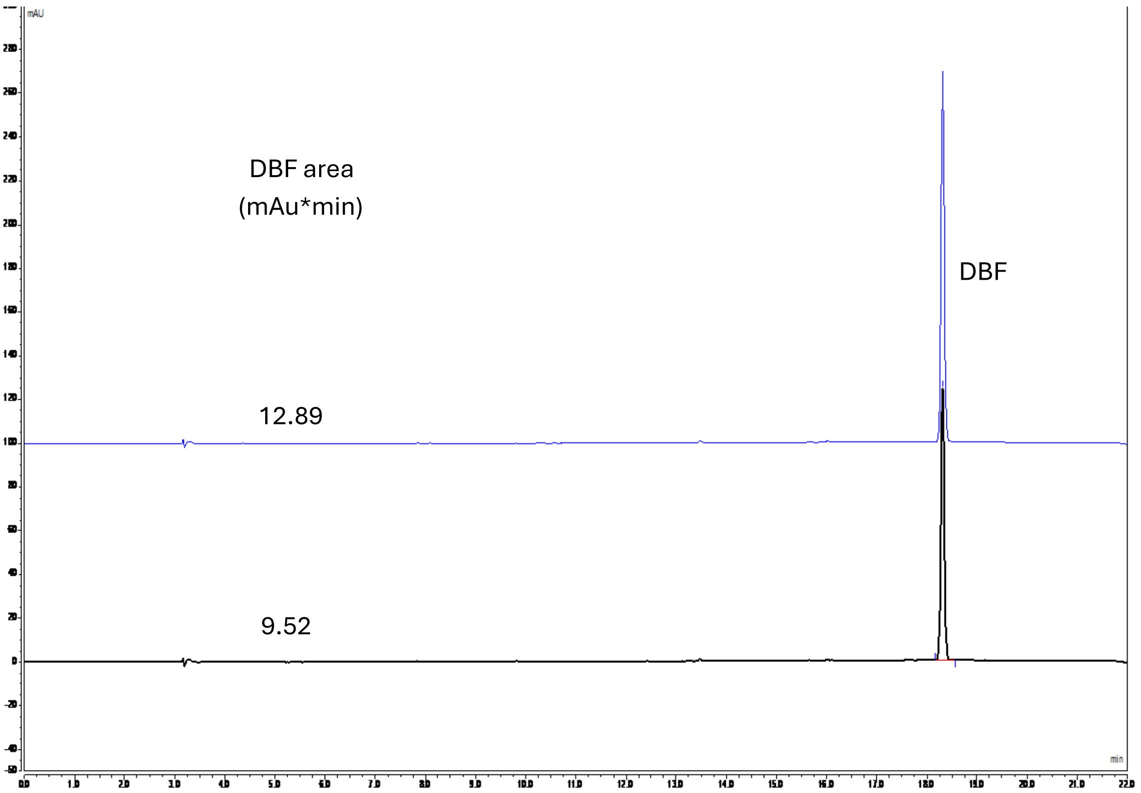

Figure S12. Overlay of LC-MS chromatograms for DBF peaks obtained by treatment of Fmoc-Leu-Sieber resin with 2% DBU in DMF; bottom, DBF peak for 0.35M Fmoc-Gly-MBH reference resin; top, DBF peak for Fmoc-Leu-Sieber resin, determined to be 0.47M by comparing the area of DBF peak of the Fmoc-Leu-Sieber resin with the DBF peak for 0.35M Fmoc-Gly-MBH reference resin.

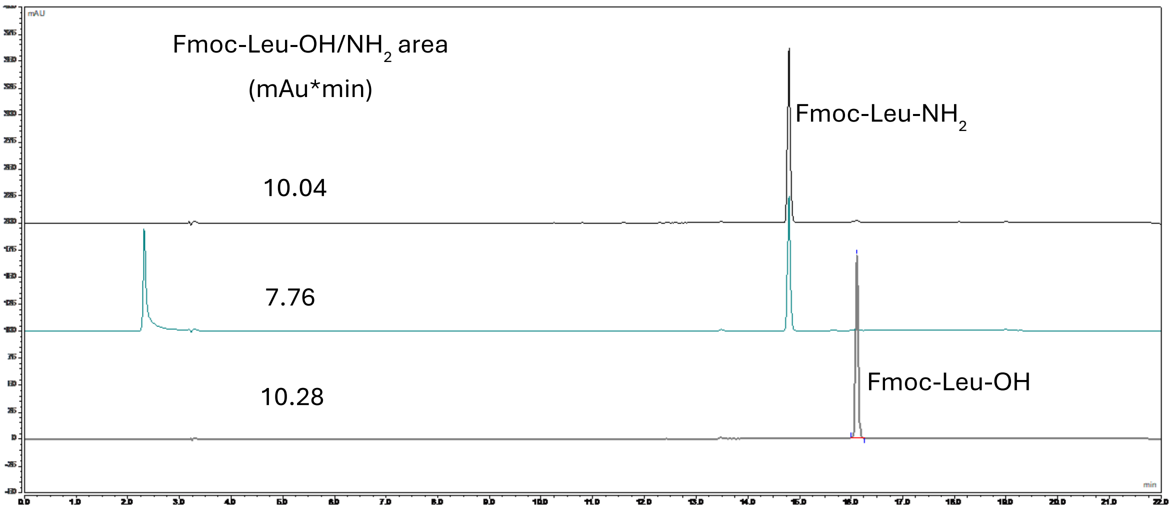

Figure S13. Overlay of LC-MS chromatograms for Fmoc-Leu-NH_2 peaks obtained by TFA in DCM and $FeCl_3$ in EtOAc cleavages of 0.47M Fmoc-Leu-Sieber; bottom to top; i. Fmoc-Leu-OH reference, 1.0 mg mL^{-1} in MeCN; ii. Fmoc-Leu-NH_2 from TFA in DCM

cleavage of Fmoc-Leu-Sieber resin; iii. Fmoc-Leu-NH_2 from $FeCl_3$ in EtOAc cleavage of Fmoc-Leu-Sieber resin.

---

[1] a) Manne, S. R.; Luna, O.; Acosta, G. A.; Royo, M.; El-Faham, A.; Orosz, G.; de la Torre, B. G.; Albericio, F. Amide Formation: Choosing the Safer Carbodiimide in Combination with OxymaPure to Avoid HCN Release. *Org. Lett.* **2021**, *23*, 6900−6904. b) Pawlas, J.; Billing, J.; Tebikachew, B.; Wahlström, L.; Haugaard-Kedström, L. M. A Sustainable Approach to ε Lys Branched GLP 1 Analogs: Integrating Green SPPS, Metal-free Alloc Removal, Waste Minimization and TFA/PFAS-free Resin Cleavage. *Org. Process Res. Dev.* **2025***, 29,* 2989–2997.

[2] Pawlas, J.; Antonic, B.; Lundqvist, M.; Svensson, T.; Finnman, J.; Rasmussen, J. H. 2D green SPPS: green solvents for on-resin removal of acid sensitive protecting groups and lactamization. *Green Chem.* **2019**, *21*, 2594−2600.

[3] Pawlas, J.; Qvist, T.; Haugaard-Kedström, L. M. Advancing Sustainable Synthesis of Cyclic Peptides by Integrating Aqueous Fmoc/t-Bu Solid-Phase Peptide Synthesis with Disulfide Bond Formation and TFA/PFAS-Free Resin Cleavage. *J. Org. Chem.* **2025**, *90*, 15909–15915.
